# Cystatin F Ensures Eosinophil Survival by Regulating Granule Biogenesis

**DOI:** 10.1016/j.immuni.2016.03.003

**Published:** 2016-04-19

**Authors:** Stephen P. Matthews, Sarah J. McMillan, Jeff D. Colbert, Rachel A. Lawrence, Colin Watts

**Affiliations:** 1 Division of Cell Signaling & Immunology, School of Life Sciences, University of Dundee, Dundee, DD1 5EH, UK; 2 Department of Comparative Biomedical Sciences, Royal Veterinary College, Royal College Street, London NW1 0TU, UK

## Abstract

Eosinophils are now recognized as multifunctional leukocytes that provide critical homeostatic signals to maintain other immune cells and aid tissue repair. Paradoxically, eosinophils also express an armory of granule-localized toxins and hydrolases believed to contribute to pathology in inflammatory disease. How eosinophils deliver their supporting functions while avoiding self-inflicted injury is poorly understood. We have demonstrated that cystatin F (CF) is a critical survival factor for eosinophils. Eosinophils from CF null mice had reduced lifespan, reduced granularity, and disturbed granule morphology. In vitro, cysteine protease inhibitors restored granularity, demonstrating that control of cysteine protease activity by CF is critical for normal eosinophil development. CF null mice showed reduced pulmonary pathology in a model of allergic lung inflammation but also reduced ability to combat infection by the nematode *Brugia malayi*. These data identify CF as a “cytoprotectant” that promotes eosinophil survival and function by ensuring granule integrity.

**Video Abstract:**

## Highlights

• Cystatin F is a novel survival factor for eosinophils
• Regulation of protease activity is critical for normal eosinophil granule biogenesis
• Parasite immunity and allergic lung pathology are reduced in cystatin F null mice

## Graphical Abstract

**Figure:**
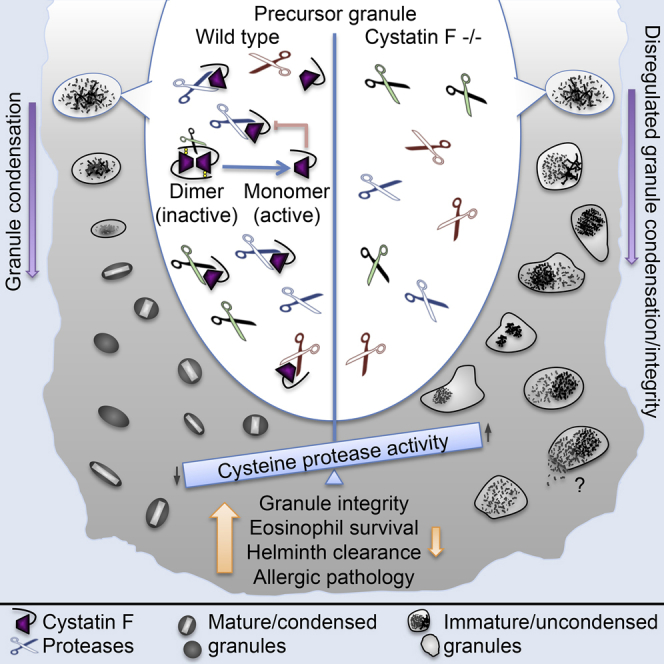

## Teaser

Eosinophils contain hallmark granules containing several toxic, basic proteins, but how they safely activate and sequester these toxins is poorly understood. In this study, Watts and colleagues show that cystatin F is critical for eosinophil viability by ensuring normal granule protein maturation through regulation of cysteine proteases.

## Introduction

Eosinophils are leukocytes that contain abundant granules whose toxic protein content provides protection against some helminths and other pathogens but additionally, and less desirably, have a deleterious impact during allergic disease. Recent reports have identified important new functions for eosinophils, which are now considered to be multifunctional leukocytes that combine cytotoxicity with key roles in maintaining other immune cells and in tissue homeostasis (Akuthota et al., 2008, Rosenberg et al., 2013). For example, long-term maintenance of plasma cells in the bone marrow depends on production of interleukin-6 (IL-6) and the B cell survival factor APRIL by eosinophils and in the gut eosinophil-derived transforming growth factor-β1 (TGF-β1) promotes antibody class switching to immunoglobulin A (IgA) and maintains gut-associated regulatory T cells and mucosal integrity (Berek, 2015). In murine adipose tissue alternatively activated macrophages are maintained by IL-4 produced by local eosinophils, whose absence led to insulin resistance and metabolic disease (Wu et al., 2011). Similarly, eosinophil-derived IL-4 is critical for muscle repair mediated by fibro/adipocyte progenitors (Heredia et al., 2013). Eosinophils have been identified as key effector cells in IL-23-driven colitis (Griseri et al., 2015) and also as professional antigen-presenting cells (Akuthota et al., 2008).

The abundant granules of eosinophils contain a battery of highly toxic proteins including major basic proteins (MBP) 1 & 2, eosinophil peroxidase (Epx), eosinophil cationic protein, and eosinophil-derived neurotoxin (Hogan et al., 2008). Upon release from granules, these proteins are toxic not only to bacterial, viral, and helminth pathogens but also to host cells and tissues (Furuta et al., 2005, Lee et al., 2004, Simon et al., 2010). Several studies link granule proteins to human eosinophil-linked diseases that affect the airways and esophagus. Taken together, the current picture presents a conundrum. How can eosinophils be both cytotoxic yet also provide a supportive environment for homeostatic processes? In particular, how does the eosinophil safely sequester its payload of toxic proteins to ensure its own survival? We have identified a survival factor for eosinophils called cystatin F.

The cystatins are naturally occurring proteins that potently inhibit the C1 family of cysteine proteases including cathepsins L, B, S, and C (Abrahamson et al., 2003, Turk et al., 2008). Cystatin F (CF), also known as “leukocystatin” because of its almost exclusive expression in immune cells, is a structurally unique member of the family being made as a disulphide-linked dimer (Cappello et al., 2004, Schüttelkopf et al., 2006). We have shown previously that dimeric CF is inactive because each sub-unit sterically inhibits the other’s ability to bind proteases (Schüttelkopf et al., 2006). In living cells, CF dimers are activated by proteolysis of the N-terminal region that links one monomer to its partner (Hamilton et al., 2008). Whereas other cystatins made with signal sequences are mostly secreted from cells, CF is targeted to the endocytic pathway (Nathanson et al., 2002) via the mannose-6-phosphate receptor (Colbert et al., 2009). The structure and mode of activation of CF suggests a unique mechanism for “buffering” vacuolar protease activity but which leukocytes might require this is not known. In this study, we generated CF null mice and report that CF is a critical survival factor for eosinophils. In its absence, granule biogenesis was abnormal and eosinophil survival was impaired. Consequently, CF null mice showed reductions in eosinophil-mediated allergic pathology and diminished protection against the nematode *Brugia malayi*.

## Results

### Absence of Normal Eosinophils in CF Null Mice

**Figure 1:**
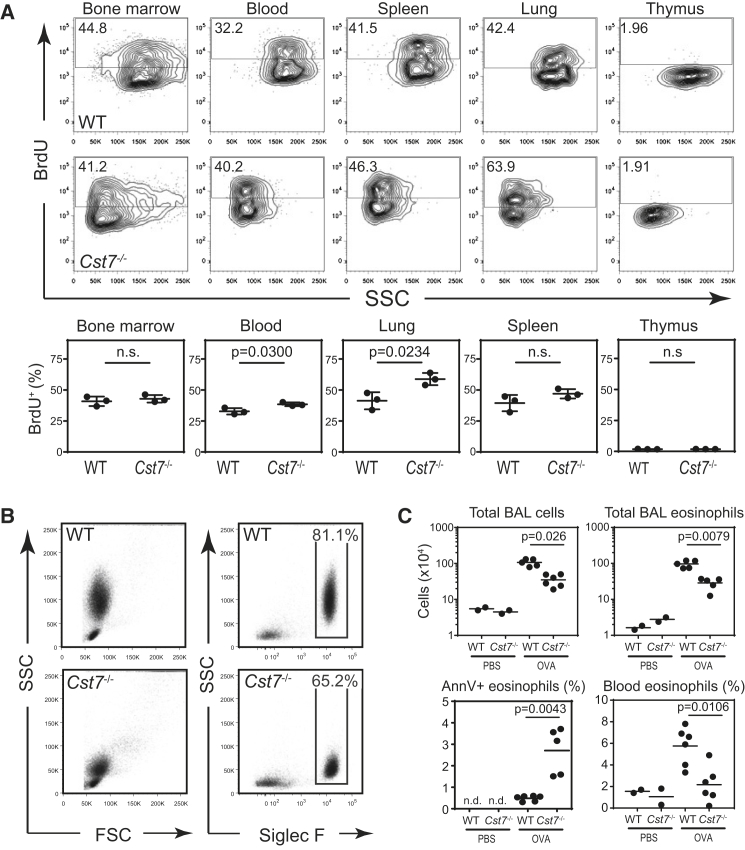
CF Null Eosinophils Have Reduced Granularity and Compromised Survival in Peripheral Tissues (A) Analysis of eosinophil granularity and turnover in naive mice. BrdU incorporation was assessed in the indicated tissues 48 hr after administration of a single dose of BrdU. Representative contour plots are shown together with means ± SD for three mice per group. (B and C) Mice were sensitized & challenged with OVA to induce acute eosinophilic lung inflammation. (B) Representative flow cytometry plots gated on total live (DAPI^−^) BAL cells from allergic mice showing absence of high SSC eosinophils. (C) Leukocyte and eosinophil accumulation in the airways and in the peripheral circulation are decreased in *Cst7*^−/−^ mice during acute allergic inflammation. The frequency of apoptotic eosinophils (Annexin V^+^, DAPI^−^) was assessed by flow cytometry and is also shown. Data represent individual mice plus means and are from 1 of 5 experiments with similar outcome. See also Figures S2 and S3.

To determine the impact of CF ablation on leukocyte populations, we generated CF null mice by loxP-mediated excision of exon 2 of the CF-encoding gene *Cst7* removing a substantial part of the coding sequence to produce a frameshift of downstream sequences (Figure S1A). Protein expression was lost in all tissues including lymph node and spleen (Figure S1B) and in the granular leukocytes where CF is most abundantly expressed including CD8^+^ and γδ T cells, neutrophils and eosinophils (Figure S1C). Gross morphology of lymphoid organs was unchanged in CF null mice. Because CF is found in leukocyte granules (Hamilton et al., 2008) we assessed not only leukocyte numbers, which appeared essentially normal in naive null mice (Figure S2), but also cell granularity as assessed by flow cytometry side-scatter (SSC). In blood, bone marrow and in other tissues we consistently found that Siglec-F^+^ eosinophils from CF null mice had very low SSC relative to wild-type (WT) eosinophils (Figure 1A and Figure S2). Although steady-state eosinophil production in the bone marrow was essentially the same in WT and CF null mice, in blood, and especially in the lung, significantly more CF null eosinophils became BrdU positive over a 48 hr period implying faster turnover of this population of granulocytes (Figure 1A).

We asked whether the accelerated turnover of CF null eosinophils in the lung might be due to impaired survival upon activation potentially leading to differences in eosinophil numbers during conditions of active eosinophilic inflammation. To test this, we used a model of ovalbumin-specific allergic lung inflammation. As expected, this led to the recruitment of large numbers of eosinophils with high SSC. These cells were almost completely absent in CF-deficient animals and were replaced by Siglec F^+^ cells with much reduced granularity (Figures 1B and S3). Moreover, under these conditions there was a dramatic reduction both in the total inflammatory cell infiltrate in the airways of CF null mice and in the accumulation of eosinophils, which was ∼70% lower than in WT mice (Figures 1C and S3). In contrast, neutrophil, dendritic cell, and other leukocyte numbers were unchanged (Figure S3). The shortfall in lung eosinophilia could not be explained by decreased eosinophil generation since even under allergic conditions there were equivalent numbers of eosinophils in the bone marrow of CF null and WT mice (data not shown). Nevertheless, blood eosinophilia increased ∼3 fold in allergic WT mice but significantly less so in CF null mice (Figure 1C). Consistent with the reduction in lung eosinophilia, we also observed a significantly higher incidence of Annexin V^+^, DAPI^−^ “early” apoptotic eosinophils in the CF null mouse lungs, indicating eosinophil death in the absence of CF (Figure 1C).

### The Requirement for CF Is Cell Autonomous

**Figure 2:**
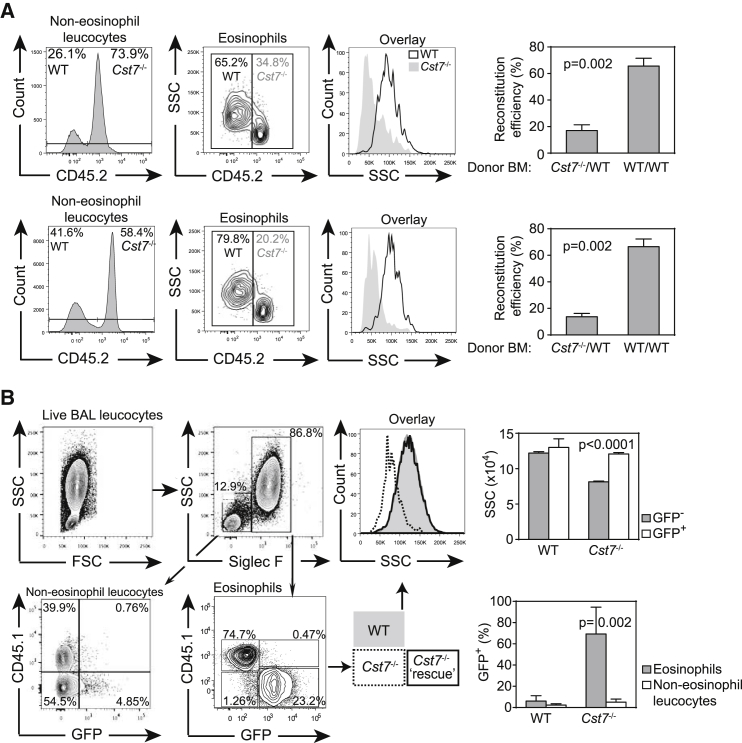
Mixed Radiation Chimerae Demonstrate a Cell Autonomous Requirement for CF in Eosinophils (A) Lethally irradiated mice were reconstituted with equal numbers of CD45.1^+^ WT and CD45.2^+^*Cst7*^−/−^ or WT bone marrow cells. Leukocyte repopulation was assessed in bone marrow (top panels) and peripheral blood (lower) 6 weeks later. Data (means ± SEM, n = 5 per group) were normalized by dividing the percent reconstitution in the eosinophil compartment by that in non-eosinophil leukocytes for each donor genotype to determine reconstitution efficiency. (B) Irradiated CD45.1^+^ hosts were reconstituted with mixed WT and *Cst7*^−/−^ donor cells infected with retrovirus expressing eGFP and CF. Five weeks later, mice were sensitized to OVA and allergic lung inflammation was induced with nebulized OVA. BAL cells were analyzed by flow cytometry and eosinophils identified as WT (CD45.1^+^) or CF null (CD45.1^−^). Reconstitution of CF (indicated by eGFP expression) rescues eosinophil numbers and completely restores granularity as assessed by SSC (histogram overlay). Few non-eosinophil CD45.2^+^ leukocytes expressed CF indicating that CF does not confer a survival advantage in other compartments. Histograms show rescue of granularity (SSC; means ± SD) and proportion of cells expressing eGFP (means ± SEM) (n = 4).

We next asked whether the phenotype was due to lack of CF in eosinophils or, for example, in another cell type that supports normal eosinophil development. We reconstituted lethally irradiated mice with equal numbers of bone marrow cells from WT (CD45.1) and CF null (CD45.2) mice. Six weeks after reconstitution, we monitored repopulation in the blood and bone marrow. As shown in Figure 2A, WT eosinophils outnumbered CF null eosinophils after reconstitution whereas other leukocytes were equally efficiently populated by WT and null cells. Moreover, eosinophils that were established from CF null marrow had much reduced SSC confirming an eosinophil-intrinsic requirement for CF (Figure 2A). To establish unequivocally that loss of CF accounted for the phenotype, we attempted to rescue normal eosinophil granularity and numbers by CF re-expression. Donor bone marrow, consisting of a mixture of WT and CF null cells, was transduced with a retrovirus expressing CF and, via an IRES element, eGFP. After reconstitution, we established allergic inflammation in the lung as described above and analyzed eGFP as a marker of CF expression in eosinophil and non-eosinophil populations. As shown in Figure 2B, less than ∼5% of WT eosinophils expressed eGFP whereas >90% of CF null eosinophils were eGFP^+^ demonstrating a very strong selection for eosinophils with restored CF expression. In contrast, few non-eosinophil leukocytes became GFP^+^ confirming that CF does not advantage other lineages (Figure 2B). In addition, although CD45.2^+^, eGFP^−^ eosinophils retained the CF null low SSC phenotype, granularity of the rescued eGFP^+^, CF^+^ fraction was completely restored to that of WT demonstrating that not only eosinophil numbers but also granularity was rescued by CF re-expression (Figure 2B).

Reduced numbers of eosinophils and abnormal granularity might indicate a developmental abnormality. However, as shown in Figure S4A, IL-5Rα^+^, c-Kit^lo^ eosinophil precursors were present in normal numbers in CF null bone marrow as were upstream granulocyte macrophage precursors. Moreover, blood eosinophil granularity was unaffected by loss of the widely expressed cystatin, cystatin C (Figure S5). Taken together, these data suggest a non-redundant requirement for CF in eosinophils at some point downstream of their expansion from bone marrow progenitors.

### The Granule Ultrastructure of CF Null Eosinophils Is Highly Abnormal

**Figure 3:**
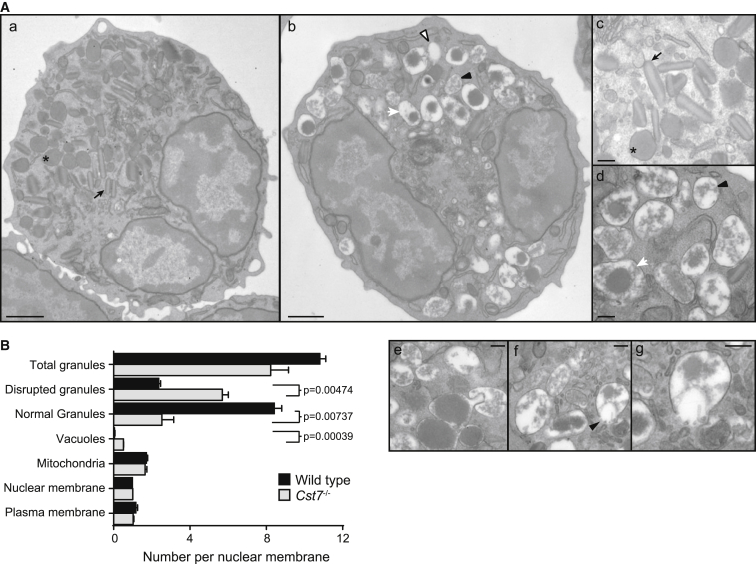
Abnormal Granule Architecture in CF Null Eosinophils (A) Electron micrographs of ex vivo bone marrow eosinophils purified from OVA-allergic WT (a and c) or *Cst7*^−/−^ mice (b, d–g). WT eosinophils contain many characteristic compact rod-like granules with electron lucent crystalline cores and a dense matrix as indicated by arrows (a and c), and also condensed granules lacking a detectable crystalline core (^∗^). Far fewer normal granules are found in CF null eosinophils (b and d), which instead contain many larger partially condensed (arrowhead) or uncondensed granules (white arrow) and, occasionally, no electron dense material at all (“vacuoles”; open arrowhead). Club-like granule extrusions as shown in (e) were frequently observed and in some cells the limiting membranes appeared incomplete suggesting possible release of granule contents (arrowhead in f and enlarged in g). (B) Quantitation of granule morphology in purified eosinophils. Condensed, electron lucent granules with or without a detectable core as described above (a and c) were considered normal; all other partially or uncondensed granule profiles with irregular or absent matrix (as in b, d–g) were considered disrupted. Data are means ± SEM of two independent experiments with sorted bone marrow cells from five pooled mice per group. At least 100 cells were counted in each of two grids per experiment. Scale bars represent 1 μm (a and b) or 0.2 μm (c–g).

To understand the cell biological basis of low granularity and truncated eosinophil lifespan in the absence of CF, we examined eosinophils purified from the bone marrow of allergic WT and CF null mice by transmission electron microscopy. As expected, WT eosinophils had numerous granules many with a distinct major basic protein (MBP)-rich crystalloid core (Popken-Harris et al., 1998) (Figures 3A and 3C). The appearance of CF null eosinophils was radically different (Figures 3A and 3B). Most cells contained large granules that had an extensive electron lucent periphery and internal material, which was sometimes condensed but was more often amorphous and unstructured (Figures 3A, 3D–3G). Only rarely did lucent granules contain a classical “bar-like” crystalloid core. In some instances, granule profiles appeared incomplete, suggesting release of granule contents into the cytosol (Figures 3A, 3F, and 3G). We also observed vacuolar structures, which were most likely either empty granules or granules that had failed to accumulate sufficient electron dense material to be detected. We quantitated granule numbers per nuclear profile distinguishing between normal condensed granules and those that appeared disrupted or otherwise unstructured. Despite these substantial differences in morphology the frequency of all granular structures was not significantly different between WT and CF null eosinophils. However, as shown in Figure 3B, WT eosinophils had 4-fold greater numbers of normal granules compared with CF null eosinophils. Reduced numbers of dense, light-scattering crystalloid granules in CF null eosinophils explains their unusual flow cytometric properties (Figure 1B). Moreover, ultrastructural profiles that indicate release of toxic granule content to the cytosol might explain reduced survival of CF null eosinophils.

### CF Is Required for Normal In Vitro Generated Eosinophils but Not Other Granulocytes

**Figure 4:**
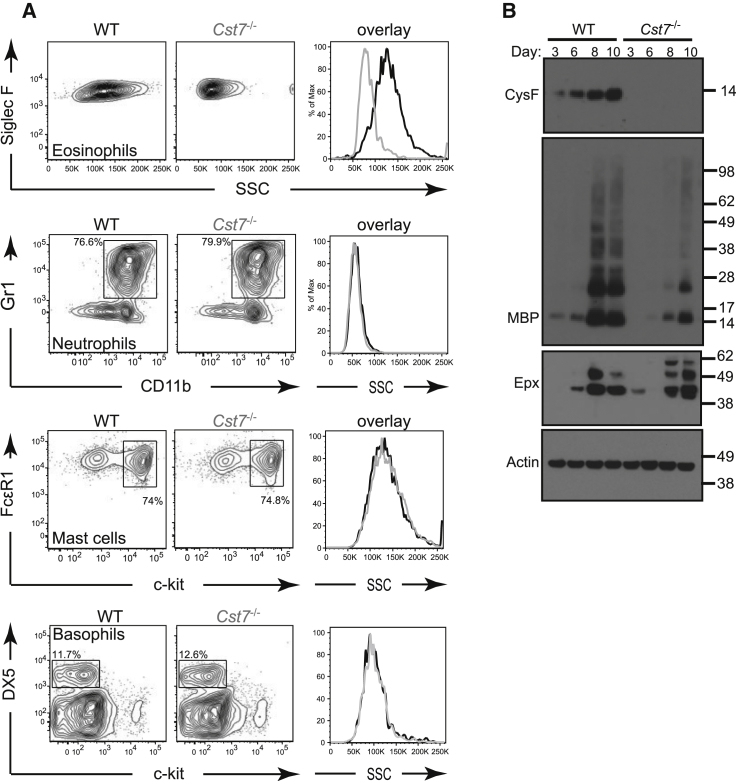
Reproduction of the CF Null Phenotype In Vitro Reduced granularity is seen in CF null eosinophils differentiated in vitro but not in other cultured granulocytes. (A) Eosinophils, neutrophils, mast cells and basophils were generated from WT or *Cst7*^−/−^ bone marrow as described in Experimental Procedures. Histogram overlays show SSC comparisons. (B) Lysates were prepared from 250,000 cells from eosinophil cultures at the indicated time points after addition of IL-5 and separated by reducing SDS-PAGE for detection of CF, MBP-1, Epx, and actin by immunoblotting. Data are representative of at least three independent experiments.

We next asked whether eosinophils differentiated in vitro from bone marrow precursors also required CF for normal granularity and survival. We adopted the protocol described by Dyer and colleagues using IL-5 to generate eosinophils from bone marrow precursors expanded in stem cell factor (SCF-1) and fms-related kinase 3-ligand (Flt3-L) (Dyer et al., 2008). Although the WT eosinophils generated were less granular than those analyzed directly ex vivo, in vitro-generated CF null Siglec F^+^ cells had an even lower SSC confirming a cell intrinsic CF requirement for normal granularity (Figure 4A). Consistent with this, and in agreement with the frequent absence of the crystalline granule core seen ultrastructurally, the granule constituent MBP-1 was much reduced relative to WT in CF null in vitro generated eosinophils (Figure 4B) although MBP-1 mRNA transcripts measured by semiquantitative PCR were essentially the same in both cell types (data not shown). Some differences in Epx processing in the CF null cells were also noted but these did not appear to affect overall Epx protein (Figure 4B) or activity relative to wild-type (data not shown). The effect of CF ablation on leukocyte granularity in vitro was specific to the eosinophil because neutrophils, basophils, or mast cells expanded from bone marrow precursors had identical SSC in CF null and WT cells (Figure 4A).

### CF Is a Cell Survival Factor for Eosinophils

**Figure 5:**
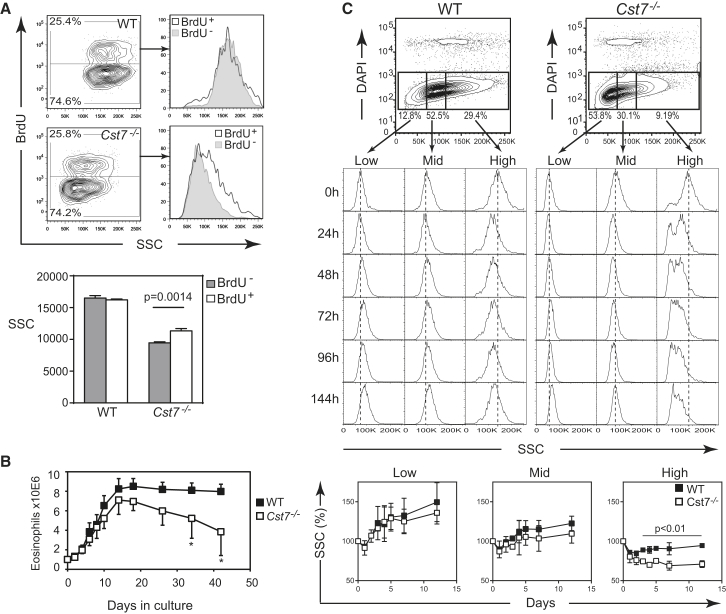
CF Stabilizes Granule Integrity and Eosinophil Viability (A) Naive mice received a single dose of BrdU 16 hr before sacrifice & assessment of incorporation into eosinophils in the bone marrow. Example flow cytometry plots are shown together with means ± SEM from two independent experiments (n = 4 per group). (B) Bone marrow cells from WT or *Cst7*^−/−^ mice were cultured for 4 days in Flt-3L and SCF-1 prior to removal of dead cells by centrifugation over Ficoll-Paque. One million viable cells were seeded into IL-5 medium and eosinophil expansion and survival was monitored by cell counting and flow cytometric identification of Siglec F^+^ cells. Means ± SD of five independent experiments are shown (^∗^p < 0.0005). (C) In vitro differentiated eosinophils were harvested after 8 days of IL-5 culture, flow cytometry-sorted into populations with high, medium, or low granularity (top panels), and chased in IL-5 culture for up to 12 days. Granularity of Siglec F^+^ cells was assessed at the indicated time points. Bottom panel shows change in granularity over the chase period (means ± SEM from two independent experiments). See also Figure S4.

The results thus far reveal a critical role for CF in maintaining eosinophil numbers and granularity but not how those two parameters might be linked. Do high-granule cells fail to develop in the absence of CF or do they develop but then have a truncated lifespan? We speculated that eosinophil granules and their constituent toxic proteins might adversely affect host cell viability and that CF could play an important role as a “cytoprotectant.” In this case, cells with a high granule content might be most vulnerable consistent with their absence when CF is lost. We tried to resolve these issues both in vivo and with the in vitro eosinophil culture system. To label newly generated eosinophils in vivo, we treated mice with a 16 hr pulse of BrdU and examined BrdU^+^ (new) and negative (older) eosinophils in bone marrow. As shown in Figure 5A, equal numbers of BrdU^+^ eosinophils were found in WT and CF null mice but the granularity of newly generated CF null eosinophils was higher than that of older, BrdU^−^ cells. In contrast, the granularity of older versus newly generated WT eosinophils was the same (Figure 5A). We next examined differentiation and survival of eosinophils generated from bone marrow in vitro. WT and CF null cultures contained similar numbers of eosinophil precursors after the 4 day expansion in Flt3-L and SCF-1 (Figure S4B). As previously described (Dyer et al., 2008), WT eosinophils were detectable within 2 days after addition of IL-5 and continued to emerge for a further 10–14 days during which time cell numbers increased 8–10 fold to comprise >95% eosinophils (Figure 5B). CF null eosinophil expansion over the same period was generally lower, but this did not reach significance, consistent with the normal rate of generation in vivo. However, over longer times there was a significant loss in viability in the CF null eosinophils (Figure 5B). To explore this further, we sorted IL-5-expanded eosinophils from WT and CF null bone marrow cultures, into low, medium, and high SSC populations and recultured them for up to 12 days in vitro. During this culture period sorted SSC^hi^ WT cells showed a small loss of granularity but CF null eosinophils exhibited a rapid, irreversible loss of highly granular cells (Figure 5C). In contrast, SSC^lo^ sorted WT cells increased their granularity over the culture period due presumably to nascent granule maturation. This occurred to a more limited extent in low granule CF null cells (Figure 5C). Taken together, these results show that a small fraction of eosinophils with more abundant granules can develop in CF null mice but do not survive without CF.

### CF Regulates Eosinophil Cysteine Protease Activity to Ensure Normal Granularity and Viability

**Figure 6:**
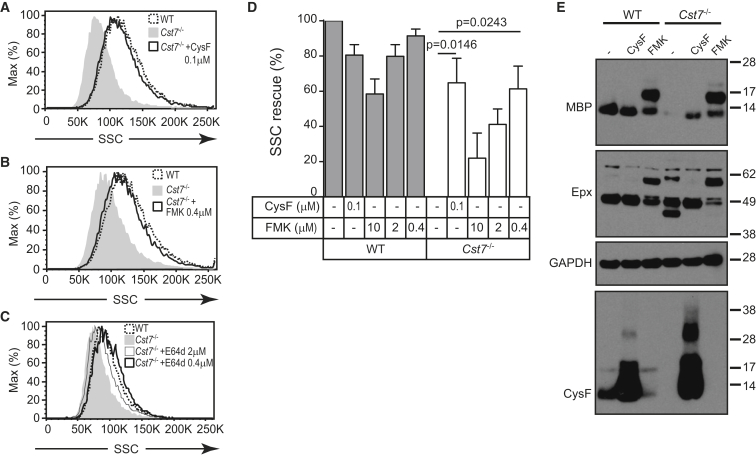
CF Null Eosinophil Granularity Can Be Stabilized by Inhibition of Cysteine Protease Activity (A–C) Protease inhibitors were added to in vitro cultured eosinophils after 4 day growth in IL-5 and replenished every other day for 9 days. Histogram overlays show increase in SSC of *Cst7*^−/−^ eosinophils treated with (A) recombinant mouse CF or with the irreversible broad-spectrum cysteine protease inhibitors z-FA-fmk (B) and E64d (C). (D) Quantitation of SSC-rescue with CF and z-FA-fmk. Data are expressed as percent restoration of the difference in mean SSC between control WT & *Cst7*^−/−^ eosinophils; means ± SD of three independent experiments are shown. (E) Lysates from 200,000 cells cultured as in (D) were separated by reducing SDS-PAGE (z-FA-fmk = 0.4 μM). MBP, Epx, CF, and GAPDH loading control were detected by immunoblot. Data are representative of at least three independent experiments.

Although other functions are not ruled out, CF likely regulates one or more granule cysteine proteases. Proteomic analysis of isolated eosinophil granules identified several cysteine cathepsins including cathepsin S, B, Z, C, and L as well as other lysosomal proteases (Table S1). If disregulated cysteine protease activity in CF null mice contributes to eosinophil granule abnormalities, we reasoned that suppression of such proteases might restore deficient granularity. In an earlier study, we have shown that recombinant dimeric CF can be taken up and activated within the endocytic pathway (Colbert et al., 2009). Indeed, inclusion of dimeric CF during eosinophil culture rescued granularity in CF null cells (Figures 6A, 6D, 6E). We also tested several small molecule protease inhibitors. These experiments were challenging because most inhibitors were toxic over the time course of eosinophil generation and had to be carefully titrated. This toxicity was specific to eosinophils as neutrophil growth was unaffected at the same concentrations (data not shown). Inclusion of the cysteine protease inhibitors z-FA-fmk or E64d resulted in a striking rescue of granularity in CF null eosinophils (Figures 6B–6D) but did not affect the granularity of WT cells. The rescue of CF null granularity was optimal at 0.4 μM for both inhibitors, which inhibited approximately 20% of cathepsin activity in the cells as measured in lysates with the broad-spectrum substrate z-Phe-Arg-AMC. Higher concentrations inhibited more protease activity but became rapidly toxic leading to cell death and, at 10 μM z-FA-fmk, a reduction in WT granularity (Figure 6D). These data strongly suggest that a primary role of CF is to regulate cysteine protease activity in developing eosinophils. The toxic effects of cathepsin inhibition indicate that finely calibrated cysteine protease activity is required for normal eosinophil development and that CF can provide such calibration. We next analyzed granule proteins in rescued cells. The low MBP-1 content in CF null eosinophils was substantially increased by culturing with dimeric CF (Figure 6E) a proportion of which was converted to the active monomer (∼14 kD). In addition, altered Epx processing in CF null eosinophils was corrected by addition of CF (Figure 6E), although the total amount of Epx protein and activity was unchanged and equivalent to WT (data not shown). Treatment with z-FA-fmk, however, prevented the complete conversion of both MBP-1 and Epx to the mature forms, suggesting that cysteine proteases participate in this conversion. Together, these data confirm that CF regulates processing of multiple granule constituents and support the notion that disturbed MBP processing is a major cause of the reduced granularity of CF null eosinophils. Furthermore, the contrasting effects of CF and z-FA-fmk upon MBP and Epx processing emphasize the importance of calibrated control of protease activity uniquely provided by CF for proper granule biogenesis.

### Reduced Eosinophil-Dependent Immunopathology and Protective Immunity in CF Null Mice

**Figure 7:**
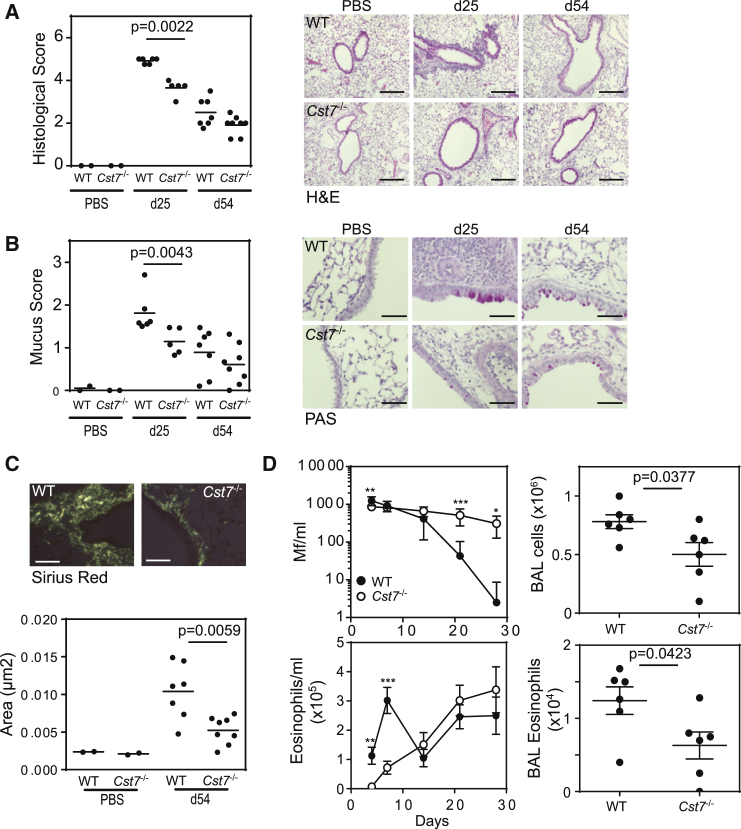
CF Null Mice Present Reduced Eosinophil-Dependent Allergic Pathology and Compromised Antihelminth Immunity (A) Mice (5–7 per treatment group) were sensitized to OVA and challenged with OVA aerosol for 5 days (acute protocol; sacrificed on day 25) or 5 weeks (chronic protocol; day 54). Pulmonary pathology was significantly reduced in CF null mice during both acute and chronic phases of allergen-induced airway inflammation as assessed by inflammatory response, mucus staining, and lung remodeling as follows: (A) H&E-stained lung sections showed decreased inflammatory infiltrate in the acute phase; (B) Periodic acid-Schiff staining indicating reduction in mucus-producing epithelial goblet cells (magenta) in the CF null airways at d25; (C) Sirius red detection of subepithelial matrix deposition visualized under polarized light. For each analysis, representative images are shown together with scores for individual mice plus group means. Scale bars represent 200 μm (original magnification ×10) for all images except panels showing mucus staining (50 μm; original magnification ×40). (D) 100,000 *B. Malayi* Mf were injected i.v. into WT (n = 6) or *Cst7*^*−/−*^ mice (n = 7) and parasite burden (Mf/ml) and blood eosinophilia were monitored by blood sampling for 4 weeks (means ± SD; p values: ^∗^ < 0.05; ^∗∗^<0.01; ^∗∗∗^<0.001). CF null mice significantly failed to clear the parasite within this window. Lungs were lavaged on day 28 for total leukocyte and eosinophil counts. Data represent individual mice plus means ± SD. See also Figure S6.

Eosinophils have been implicated as key leukocytes in a variety of immune processes with beneficial or pathological outcomes. We therefore assessed the impact of loss of CF in a paradigm of each condition: allergic inflammation and parasite clearance. First, because eosinophils contribute to airway remodeling in some mouse models of asthma (Humbles et al., 2004, Lee et al., 2004) and to pathology in some types of human asthma (Haldar et al., 2009, Nair et al., 2009), we extended our allergic lung inflammation studies in CF null mice. During the acute phase of allergen-induced airway inflammation (day 25), peribronchiolar infiltration of eosinophils (Figure 7A) and mucus production by hyperplastic goblet cells (Figure 7B) were significantly reduced in CF null mice compared to WT. Moreover, the increase in sub-epithelial matrix deposition observed during the subsequent chronic phase of inflammation (days 25–54) was also significantly attenuated in CF null lungs (Figure 7C). Eosinophils contribute to the local cytokine milieu enhancing T helper-2 (Th2) cell responses (Spencer and Weller, 2010). In acutely allergen-exposed CF null mice several inflammatory cytokines including tumor necrosis factor-α (TNF-α), interferon-γ (IFN-γ), and IL-13 were substantially reduced as was Epx activity (Figure S6A). Taken together, these results show that loss of an eosinophil granule-localized survival factor leads to attenuated inflammation and pathological remodeling of the lung.

Finally, we tested the ability of CF null mice to expel the human nematode parasite *Brugia malayi*, a major cause of lymphatic filariasis. Recent studies using eosinophil-deficient mice have demonstrated a role for eosinophils in protection against primary infection with *B. malayi* microfilariae (Mf) (Cadman et al., 2014). We infected mice with *B. malayi* Mf and followed clearance by blood sampling. WT mice rapidly eliminated the infection, with Mf burden reduced to undetectable numbers after 28 days in 5 of 6 mice (Figure 7D). In contrast, CF null mice failed to clear the parasite during this period and this correlated with a delay in elevated blood eosinophilia (Figure 7D). Mf entrapment in the pulmonary microvasculature leads to eosinophilic lung inflammation (Cadman et al., 2014), yet despite the persistence of Mf in CF null mice upon sacrifice at day 28 of infection there was a significant reduction in total leukocytes, eosinophils, and Epx activity in the lung compared to WT (Figures 7D and S6B). Nonetheless, CF null mice also showed increased IFN-γ and Mf-specific IgE in both sera and BALF at this time point, presumably stimulated by continued presence of the parasite (Figure S6B). These data demonstrate the importance of maintaining granule integrity for efficient expansion of eosinophils during helminth infections. Further, they show that CF-mediated control of cysteine protease activity is an essential component of this maintenance without which parasite clearance is compromised.

## Discussion

Our results have identified CF as a survival factor for eosinophils. The beneficial effects of CF were most evident in eosinophils with high granule content, a sub-population that was essentially absent in CF null mice. CF is made as an inactive dimer and is converted to the active monomeric state by proteolysis (Hamilton et al., 2008). This unique mode of activation, coupled with its targeting to the endolysosomal pathway (Colbert et al., 2009), suggests that its function is to “buffer” protease activity in this domain. We propose that as protease activity rises, for example due to granule condensation and/or lower pH, more CF dimers will be converted to active monomers thus normalizing protease activity. Our data show that this tightly controlled regulation of cysteine proteases is critical for normal eosinophil granule biogenesis and eosinophil viability, due at least in part to stabilization of MBP which constitutes the granule core.

Despite the substantial loss of granule integrity, eosinophil numbers in naive mice were not significantly affected by the absence of CF. Although BrdU labeling revealed accelerated eosinophil turnover in CF null lungs, this did not result in a detectable reduction in cell numbers, presumably because steady-state eosinophil development in the bone marrow is sufficient to maintain the low frequency of eosinophils in naive lungs. Our observation that CF null eosinophils fail to accumulate normally during allergic inflammation is in agreement with studies showing that tissue eosinophilia arises due to delayed eosinophil apoptosis in response to survival signals such as IL-5 (Simon et al., 1997). In fact, one consequence of activation of eosinophils with IL-5 is rapid granule acidification (Persson et al., 2002), which would be expected to lead to increased cysteine protease activity. In the absence of CF, eosinophils might be unable to adequately respond to anti-apoptotic cues, leading to the dramatic reduction in allergen-induced eosinophilic inflammation and airway remodeling seen in null mice.

An important question concerns the identity of the proteases targeted by CF in eosinophils. We reasoned that if unregulated activity of a single cathepsin is responsible for the phenotype in CF null eosinophils, genetic deletion of this enzyme might rescue it. However, we have crossed the CF null mice with mice lacking individual cathepsins B, S, L, and C but have not as yet observed a rescue of eosinophil granularity. Most likely, CF regulates multiple proteases to stabilize granule formation.

Although CF is expressed in other granule-containing leukocytes such as neutrophils and mast cells, to date the requirement for CF appears to be eosinophil specific, at least as far as normal granularity is concerned. It will be of interest to determine whether the functional properties of other immune cell lineages are altered by loss of CF. Loss of CF resulted in dramatic changes to the appearance of eosinophil granules but the precise events in granule maturation that are disrupted remain to be characterized. To date, few studies have examined the molecular biology of granule maturation in eosinophils. MBP is synthesized as a pro-form containing a highly acidic N-terminal portion that is thought to protect the developing eosinophil from the mature basic toxin during granule biogenesis (Barker et al., 1991). Conversion to the mature form, by as yet unidentified proteases, occurs within the granule itself (Popken-Harris et al., 1998) and coincides with the formation of inert MBP nanocrystals, which repress its intracellular toxicity (Soragni et al., 2015). During degranulation, granule acidification leads to crystal disassembly enabling deposition of smaller highly cytotoxic MBP aggregates (Soragni et al., 2015). The abnormal granules we observed in CF null bone-marrow eosinophils were reminiscent of immature granules observed in developing eosinophils (Popken-Harris et al., 1998) perhaps indicative of a failure to develop properly in the absence of CF.

Other studies have described abnormal eosinophil granularity but none have replicated the phenotype of CF null eosinophils. Mice lacking MBP-1 (*Prg2*^*−/−*^) or Epx (*Epx*^−/−^) have been generated and their eosinophil granules shown either to lack the crystalline core or to contain less matrix respectively (Denzler et al., 2000, Denzler et al., 2001). In contrast to our results, eosinophil accumulation and airway histopathology during OVA-induced allergic inflammation was unaffected in these genetically deleted mice. This important difference shows that disrupted eosinophil granule morphology does not necessarily impair eosinophil viability but rather that precise regulation of cysteine protease activity during eosinophil development and activation is critical for survival. Similarly, loss of either of these granule proteins alone does not impair protection during primary infection with *B. malayi* Mf (Cadman et al., 2014). The compromised clearance of this pathogen seen in CF null mice is likely due to a combination of defective granule integrity and the failure of eosinophils to rapidly accumulate. Loss of both MBP-1 and Epx together leads to virtual ablation of the eosinophil lineage due to impaired eosinophil precursor development or survival (Doyle et al., 2013). In contrast, loss of CF did not affect eosinophil precursor viability, but rather altered MBP and Epx processing, which appears to be critical for normal granule biogenesis and biological function.

Although expression of CF is mostly confined to immune cells its expression in one other instance is noteworthy. An examination of genes differentially expressed in metastatic versus non-metastatic murine liver tumors has identified a gene which was named “cystatin-like metastasis associated protein” or CMAP (Morita et al., 1999). CMAP expression correlates with metastatic activity and tumor volume but its mechanism of action has not been determined. CMAP is identical to CF raising the possibility that some transformed cells upregulate CF expression to take advantage of its cytoprotective properties. Lysosomal cathepsins can promote apoptotic cell death via the lysosomal membrane permeabilization (LMP) pathway and there is renewed interest in selectively inducing this pathway in tumor cells (Petersen et al., 2013). Elevated CF expression in tumors might counteract LMP driven cell death by reducing the activity of lysosomal cysteine proteases.

Our results extend the concept that cytotoxic leukocytes need to protect themselves from their own toxic granules. Recent studies have shown that two members of the serpin family of serine protease inhibitors, Spi6 and Spi2a, are critical for normal cytotoxic T lymphocyte (CTL) expansion and survival of memory cells respectively. Loss of Spi6 leads to self-inflicted death of CTL through granzyme B leakage into the cytosol reducing normal CTL expansion (Zhang et al., 2006). In contrast, although initial expansion of CTL is normal in mice lacking Spi2a, numbers of memory CD8^+^ T cells are much reduced due to cathepsin B mediated programmed cell death (Liu et al., 2004). Unlike CF, these serpins are located in the cytosol. Our results reveal a topologically distinct mode of protection that operates in the lumen of eosinophil granules and possibly other granule containing leukocytes. Whereas the serpins act to suppress the pro-apoptotic effects of proteases that leak from CTL granules, CF acts to prevent granule instability by ensuring normal granule biogenesis. Thus leukocytes use two distinct families of protease inhibitors, the serpins and the cystatins, to protect themselves from self-inflicted lethal hits.

## Experimental Procedures

### Mice

CF null mice were generated (TaconicArtemis) by deletion of exon 2 of the CF-encoding gene *Cst7* as outlined in Figure S1A on a C57BL/6 background. Mice were bred and maintained under specific pathogen-free conditions and were used between 6 weeks and 6 months of age. *Cst7*^−/−^ breeding performance and litter size were comparable to WT. All animal experiments were approved by the University of Dundee Ethics Committee and were performed under a United Kingdom Home Office Project License.

### Reagents

Polyclonal antisera specific for mouse MBP-1 and Epx were generated by immunizing rabbits with synthetic peptides coupled to KLH (Scottish National Blood Transfusion Service) and affinity purified. The immunizing peptides were MBP: GGRIKGWGRC and Epx: CSRIPKLNLSAWRGK.

### Parasite Infection Protocol

Groups of six to seven, 10-week-old male mice were intravenously injected with 10^5^ *B. malayi* Mf as previously described (Cadman et al., 2014). Parasitaemia and blood counts were monitored by blood sampling 4, 7, 14, 21, and 28 days after infection. Upon termination airways were sampled by bronchoalveolar lavage (BAL).

### Ovalbumin-Induced Airway Inflammation

For induction of acute airways inflammation, mice were primed by intraperitoneal injection of 10 μg OVA (Sigma) in 100 μl Imject Alum (Thermo Scientific) on days 0 and 11 followed by daily challenge with an aerosol of 1% OVA from days 20–24 & sacrifice on day 25. Induction of chronic inflammation and lung remodeling was identical except that from day 25 mice received additional challenge with nebulized OVA 3 times per week for 4 weeks before sacrifice 24 hr after the last OVA aerosol on day 54. Airways were sampled by BAL or inflated with 4% isotonic paraformaldehyde for histology.

### Histology

4 μm paraffin-embedded lung sections (Veterinary Diagnostic Services, University of Glasgow) were stained with hematoxylin and eosin (H&E) to evaluate general morphology and with Periodic acid-Schiff (PAS) to identify epithelial goblet cells. Matrix deposition was assessed on Sirius red-stained sections. Slides were examined in a random blinded fashion. Leukocytic lung infiltration was graded with a semiquantitative scoring system on a scale of 0–5, with 1 signifying a small number of inflammatory foci and 5 a large (>3 cells deep) widespread infiltrate around the majority of vessels and bronchioles. PAS-stained goblet cells were scored as described (McMillan et al., 2002).

Sirius red staining was visualized under polarized light. Digital photographs of at least five bronchioles per slide were quantitated with Volocity V6.3.0 (Perkin Elmer) using automatic detection and measurement of stained regions. Data are expressed as the area of staining per μm length of basement membrane.

### Flow Cytometry

All analytical flow cytometry was performed on a BD LSR II flow cytometer. Dead cells were identified by DAPI (4’,6-diamidino-2-phenylindole) and excluded from analysis where appropriate. Primary antibodies specific for the following markers were directly conjugated to FITC, PE, APC, PerCP-Cy5.5, eFluor-780, eFluor-450, or PE-Cy7 and purchased from eBioscience (CD3, CD4, CD8α, CD11b, CD16/32, CD34, CD45.1, CD45.2, CD45R(B220), CD49b(DX5), FcεR1 and Gr1) or from BD Biosciences (c-kit, IL-5Rα, Siglec F, CCR3, and Annexin V). For intracellular staining, surface-labeled cells were fixed in isotonic 3% paraformaldehyde and incubated with affinity-purified anti-CF (Hamilton et al., 2008) in Fix & Perm (Invitrogen) according to product instructions. Staining was detected with highly cross-adsorbed donkey anti-rabbit-FITC (Jackson Immunochemicals, Stratech, UK). For cell sorting experiments, eosinophil populations were sorted on a BD Influx flow cytometer.

### BrdU Labeling In Vivo

Mice were injected intraperitoneally with a single dose of 100 μg BrdU 16 or 48 hr prior to sacrifice. Single cell suspensions were stained with the relevant surface antigens and BrdU incorporation was revealed using the APC BrdU Flow Kit (BD PharMingen).

### In Vitro Granulocyte Culture

Eosinophils were differentiated as described (Dyer et al., 2008) with some modifications. Briefly, bone marrow cells were depleted of erythrocytes by osmotic lysis and cultured at 10^6^ cells/ml in R20F (RPMI containing 20% FCS, 100 IU/ml penicillin, 10 μg/ml streptomycin, 2 mM glutamine, 1× non-essential amino acids, 1 mM sodium pyruvate (Life Technologies), and 50 μM β-mercaptoethanol), supplemented with 100 ng/ml stem cell factor (SCF-1) (eBioscience), and 100 ng/ml Flt-3L (Peprotech). On day 4 cells were centrifuged at 800 g for 25 min over Ficoll-Paque (GE Healthcare, UK) to remove dead cells and pre-existing granulocytes. Live cells were collected from the interface, washed, and cultured at 10^6^/ml in R20F containing 20 ng/ml recombinant mouse IL-5 (eBioscience), with media refreshed every 3 or 4 days. Other granulocyte populations were generated identically to day 4 after which enriched precursor cells were cultured in R20F plus 10ng/ml G-CSF for 6 days (for neutrophil generation) or 20 U/ml mIL-3 for up to 25 days (for basophil and mast cell differentiation). For rescue experiments, recombinant mouse CF or the inhibitors z-FA-fmk and E64d (Sigma) were added to eosinophil cultures after 4 days expansion in IL-5. Every two days half the medium was replaced with the same volume of fresh inhibitor at the same concentration.

### Immuno Blotting

Eosinophils were lysed in 10 mM Tris (pH7.4) containing 150 mM NaCl, 1 mM EDTA, 1 mM EGTA, 0.5% CHAPS, and 0.1% NP-40. Samples were separated by reducing SDS-PAGE on 10% Bis-Tris gels (Invitrogen), transferred to nitrocellulose membranes and blocked and probed in 5% milk powder in PBS/0.05% Tween-20. Rabbit antibodies and actin-specific C4 were detected with peroxidase-conjugated secondary antibodies (Jackson) and Immobilon chemiluminescent substrate (Millipore).

### Transmission Electron Microscopy

Single cell suspensions were prepared from bone marrow of OVA-immunized and challenged mice and depleted of irrelevant leukocytes using anti-rat IgG beads (Dynal Invitrogen) and antibodies specific for CD3, CD4, CD8, Ter119, and B220. The remaining cells were labeled with anti-Siglec F and anti-Gr1 and viable eosinophils (Siglec F^+^, Gr1^lo^, DAPI^–^) were enriched by flow cytometry to greater than 95% purity, fixed in 2% glutaraldehyde, 0.1M sodium cacodylate, pH 7.3, and sections mounted on electron microscopy grids. Images were recorded on a JEOL 1200 EX (80kV) with phospho-imaging plates (DITABIS, Pforzheim). For quantitation, multiple fields were selected by random sampling under high magnification (×40,000) and total numbers of nuclear membrane profiles, plasma membrane profiles, and subcellular organelles were counted in each field. At least 100 nuclear membrane profiles were counted per sample and data were normalized by dividing the total of each organelle by the number of nuclear membrane profiles counted.

### Radiation Chimerae and Retroviral Infections

For mixed bone marrow radiation chimerae, host mice were irradiated with 2 × 6 Gy delivered 3 hr apart and reconstituted by intravenous injection of 2 × 10^6^ donor cells comprising equal numbers of freshly isolated WT and *Cst7*^*−/−*^ bone marrow. WT donor cells were derived from congenic C57BL/6 mice expressing CD45.1 to enable discrimination from CD45.2^+^ *Cst7*^*−/−*^ cells in the reconstituted chimerae.

In some experiments, donor cells were infected with retroviruses expressing mouse CF and GFP. CF was cloned from spleen cDNA and inserted into the Moloney murine leukemia virus (MoMLV)-based pBMN-I-eGFP retroviral vector (provided by G. Nolan, Stanford) (Hamilton et al., 2008). Virus was produced by transfection of Phoenix Eco 293T packaging cells as previously described (West et al., 2004). Donor mice were injected intraperitoneally with 5 mg 5-fluoro-uracil and bone marrow cells were collected 3 days later, incubated overnight in culture media containing 10 ng/mL IL-3, 10 ng/mL IL-6, and 50 ng/mL SCF-1 (Peprotech), and infected with retrovirus in 5 μg/ml polybrene by centrifugation at 1,000 g × 90 min. Infection was repeated following overnight culture and plates incubated for a further 4 hr before counting cells, mixing WT and *Cst7*^*−/−*^ and injecting into irradiated donor mice.

### Eosinophil Peroxidase Assay

Epx activity was measured essentially as described (Clark et al., 2004) using 12 mM O-phenylene diamine in 10 mM HEPES pH 8 containing 0.25% cetyl-tetraethylammonium bromide and 0.01% hydrogen peroxide. 50 μl substrate solution was added to an equal volume of BAL supernatant or eosinophil lysate for 20 min at 21°C. Reactions were terminated with 5% phosphoric acid and absorbance measured at 490 nm.

### Data Analysis

For repeated-measurements in the parasite expulsion studies, two-way ANOVA was used to test for effects between groups. All other data were analyzed using the unpaired t test or, for animal experiments, the Mann-Whitney U test (Prism 6, Graphpad Software). Statistical significance was accepted when p < 0.05.

## Author Contributions

Conceptualization, S.P.M. and C.W.; Methodology, S.P.M., S.J.M., R.A.L., and C.W.; Investigation, S.P.M., S.J.M, J.D.C., and R.A.L.; Writing – Original Draft, S.P.M. and C.W.; Writing – Review & Editing, S.P.M. and C.W.; Funding Acquisition, C.W.

## Supplemental Information

Document S2. Table S1

Document S3. Article plus Supplemental Information
