# Supplementary figures and images for: Cystatin F Ensures Eosinophil Survival by Regulating Granule Biogenesis
Source: Immunity. 2016 Apr 19;44(4):795–806. doi: 10.1016/j.immuni.2016.03.003 (PMC4846977; doi:10.1016/j.immuni.2016.03.003)

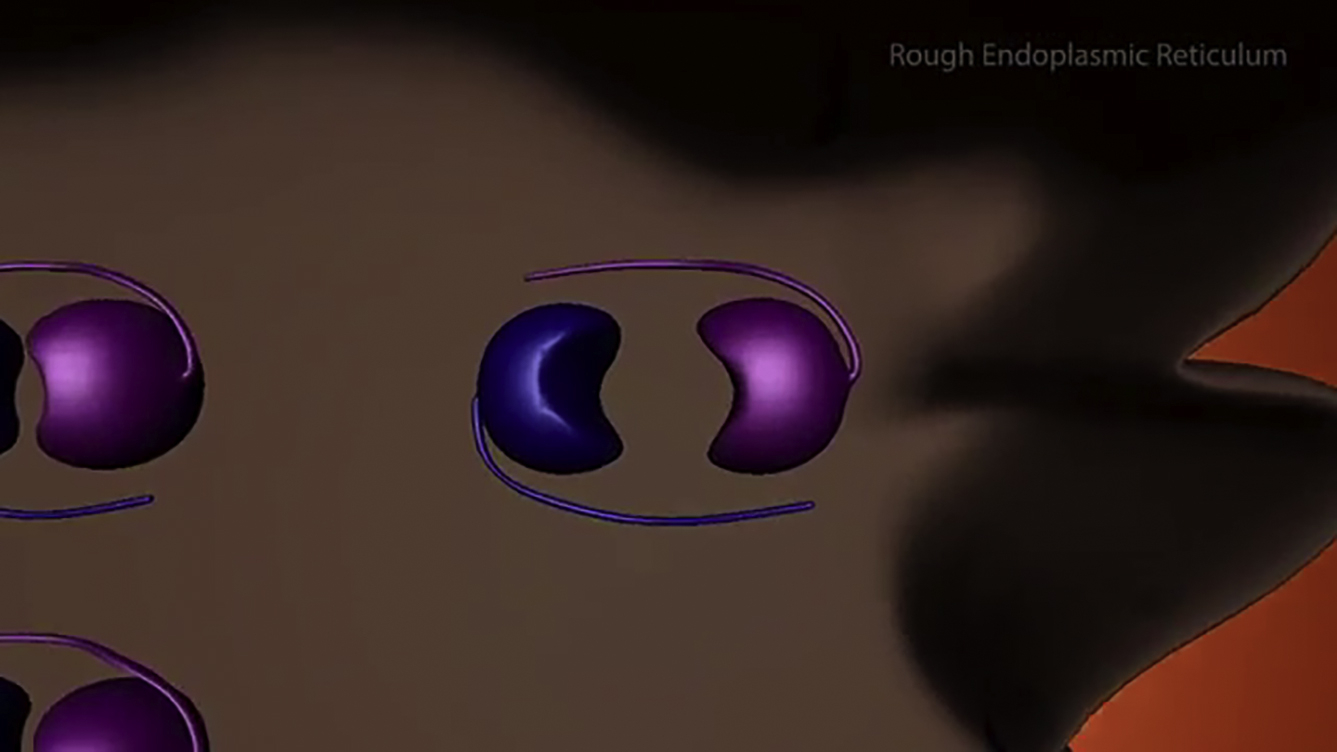

Supplement: Supplementary file 1 [file mmc4.jpg]
