# Supplementary material for: Cystatin F Ensures Eosinophil Survival by Regulating Granule Biogenesis
Source: Immunity. 2016 Apr 19;44(4):795–806. doi: 10.1016/j.immuni.2016.03.003 (PMC4846977; doi:10.1016/j.immuni.2016.03.003)
Supplement: Document S1. Figures S1–S6 [file mmc1.pdf]

Immunity, Volume *44*

Supplemental Information

Cystatin F Ensures Eosinophil Survival by Regulating Granule Biogenesis

Stephen P. Matthews, Sarah J. McMillan, Jeff D. Colbert, Rachel A. Lawrence, and Colin Watts

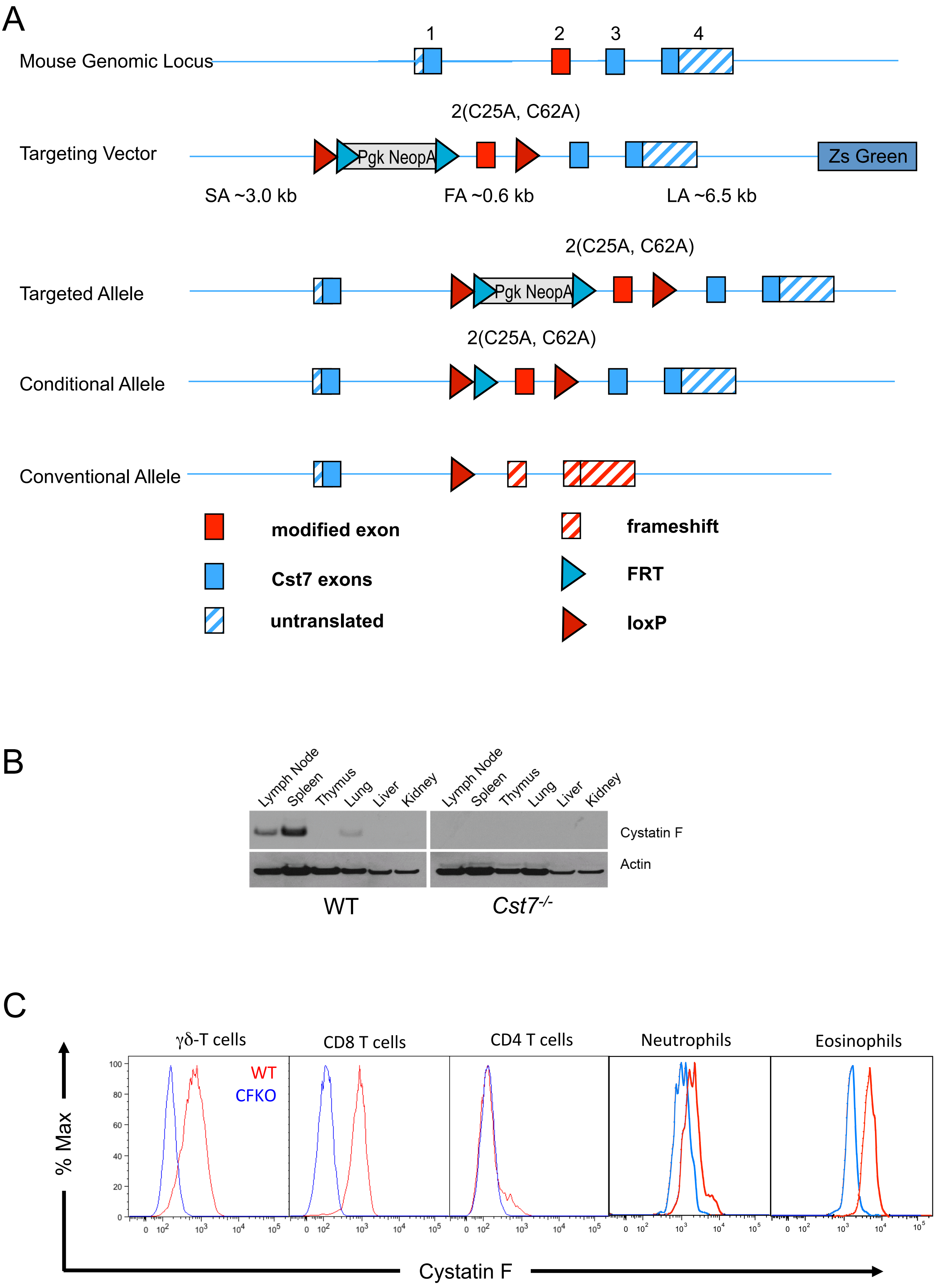
A
1
2
3
4
Mouse Genomic Locus
2(C25A, C62A)
Targeting Vector
Pgk NeopA
Zs Green
SA ~3.0 kb
FA ~0.6 kb
LA ~6.5 kb
2(C25A, C62A)
Targeted Allele
Pgk NeopA
2(C25A, C62A)
Conditional Allele
Conventional Allele
modified exon
Cst7 exons
untranslated
frameshift
FRT
loxP
B
Lymph Node
Spleen
Thymus
Lung
Liver
Kidney
Cystatin F
Actin
WT
Cst7-/-
C
γδ-T cells
CD8 T cells
CD4 T cells
Neutrophils
Eosinophils
WT
CFKO
% Max
Cystatin F

Figure S1

Figure S1, related to Experimental Procedures: Generation and characterization of CF-null mice (*Cst7-/-*).
(A). Overview of targeting strategy for generation of CF-null mice.
B, C: Confirmation of CF deletion in *Cst7-/-* mice. (B) Whole tissue lysates were prepared from the indicated organs, separated by reducing SDS-PAGE and immunoblotted for detection of cystatin F and β-actin. CF protein is expressed in lymph node, spleen & lung of wild type control but not *Cst7-/-* mice. (C) Intracellular staining & flow cytometric detection of CF in the indicated cell subsets directly ex vivo. CF is specifically expressed in wild type leucocytes (red) that elaborate specialized exocytic granules (CD8+ T cells, γδ T cells, neutrophils & eosinophils) but not in CD4+ T cells. Expression is lost in *Cst7-/-* cells (blue).

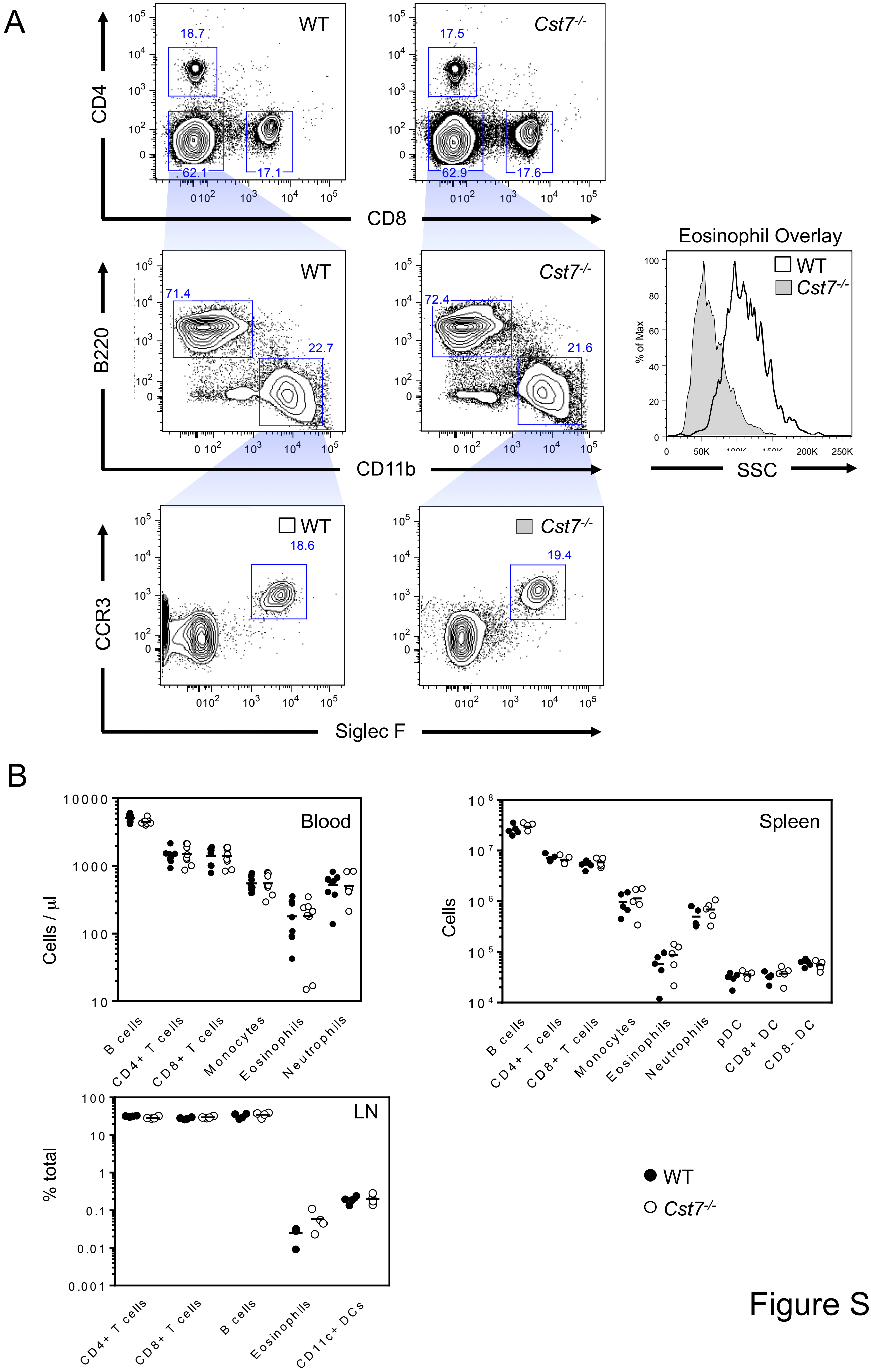
A
WT
Cst7-/-
CD4
CD8
18.7
62.1
17.1
17.5
62.9
17.6
B220
CD11b
71.4
22.7
72.4
21.6
Eosinophil Overlay
% of Max
SSC
CCR3
Siglec F
18.6
19.4
B
Blood
Cells / µl
B cells
CD4+ T cells
CD8+ T cells
Monocytes
Eosinophils
Neutrophils
Spleen
Cells
pDC
CD8+ DC
CD8- DC
LN
% total
CD11c+ DCs

Figure S2

Figure S2, related to Figure 1: Phenotypic analysis of leucocyte populations in CF null mice.

Peripheral blood leucocyte subsets were analysed by flow cytometry. (A) Typical gating strategy for identification of white blood cells including eosinophils ($CD11b^+$ $CCR3^+$ Siglec F^+). (B) Counts for the indicated leucocyte populations in peripheral blood, spleen & brachial lymph node. Values for individual mice plus means for each group are shown.

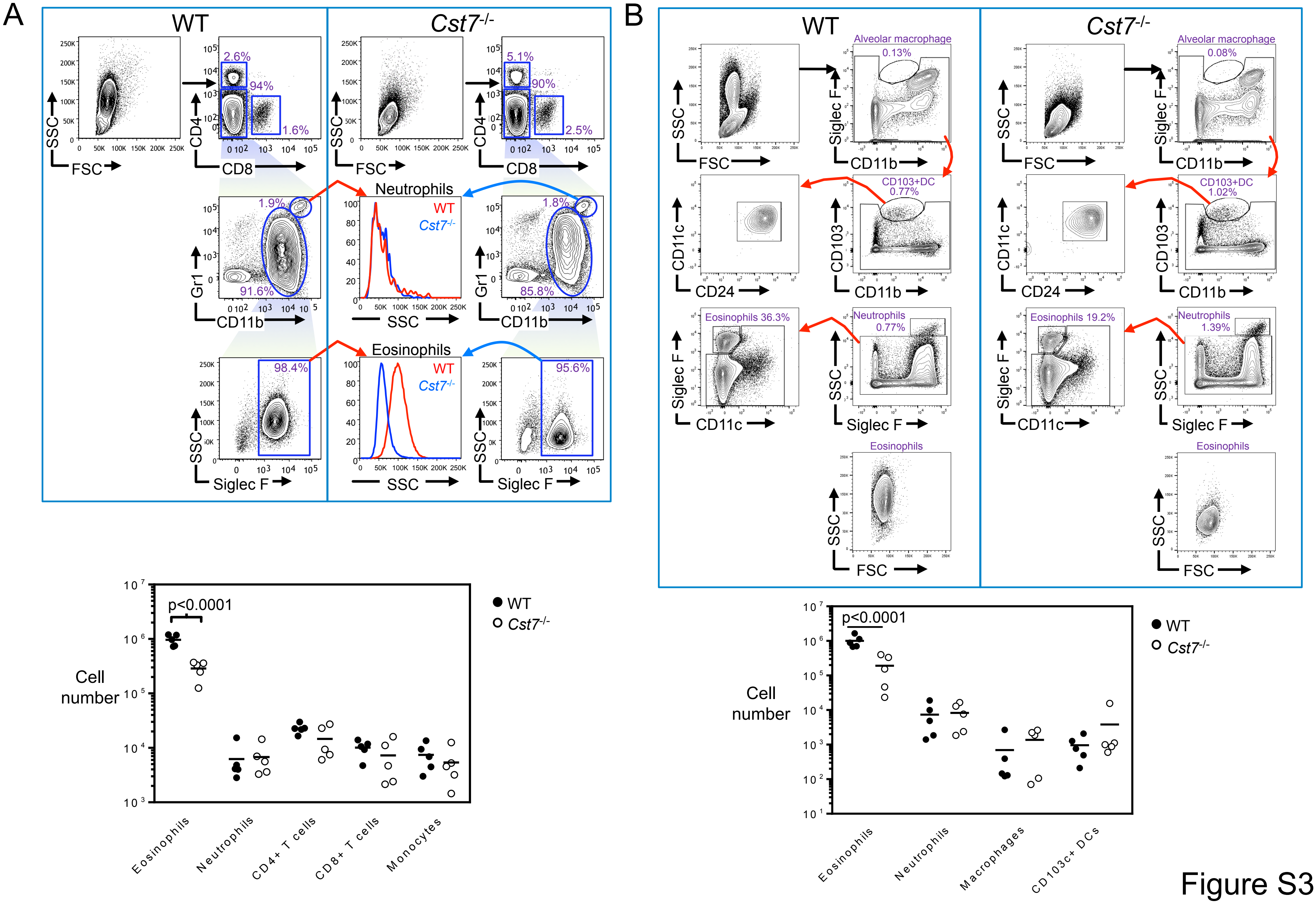
A
WT
Cst7-/-
SSC
FSC
CD4
CD8
2.6%
94%
1.6%
5.1%
90%
2.5%
Gr1
CD11b
1.9%
91.6%
1.8%
85.8%
Neutrophils
Eosinophils
WT
Cst7-/-
Siglec F
98.4%
95.6%
B
Alveolar macrophage
0.13%
0.08%
CD103+DC
0.77%
1.02%
CD11c
CD24
CD103
Eosinophils 36.3%
Eosinophils 19.2%
Neutrophils
0.77%
1.39%
Eosinophils
Cell number
p<0.0001
Neutrophils
CD4+ T cells
CD8+ T cells
Monocytes
Macrophages
CD103c+ DCs

Figure S3

Figure S3, related to Figure 1: Phenotypic analysis of leucocyte subsets in allergic lungs.
(A) FACS staining strategy for identification of major leucocyte subsets in the BALF of ova-sensitized mice after 5d of ova-aerosol. Lower panel: total cell counts (values for individual mice plus means).
(B) FACS staining strategy for identification of alveolar macrophages and tissue dendritic cells in whole lung digests. Lower panel: cell numbers for individual mice plus means.

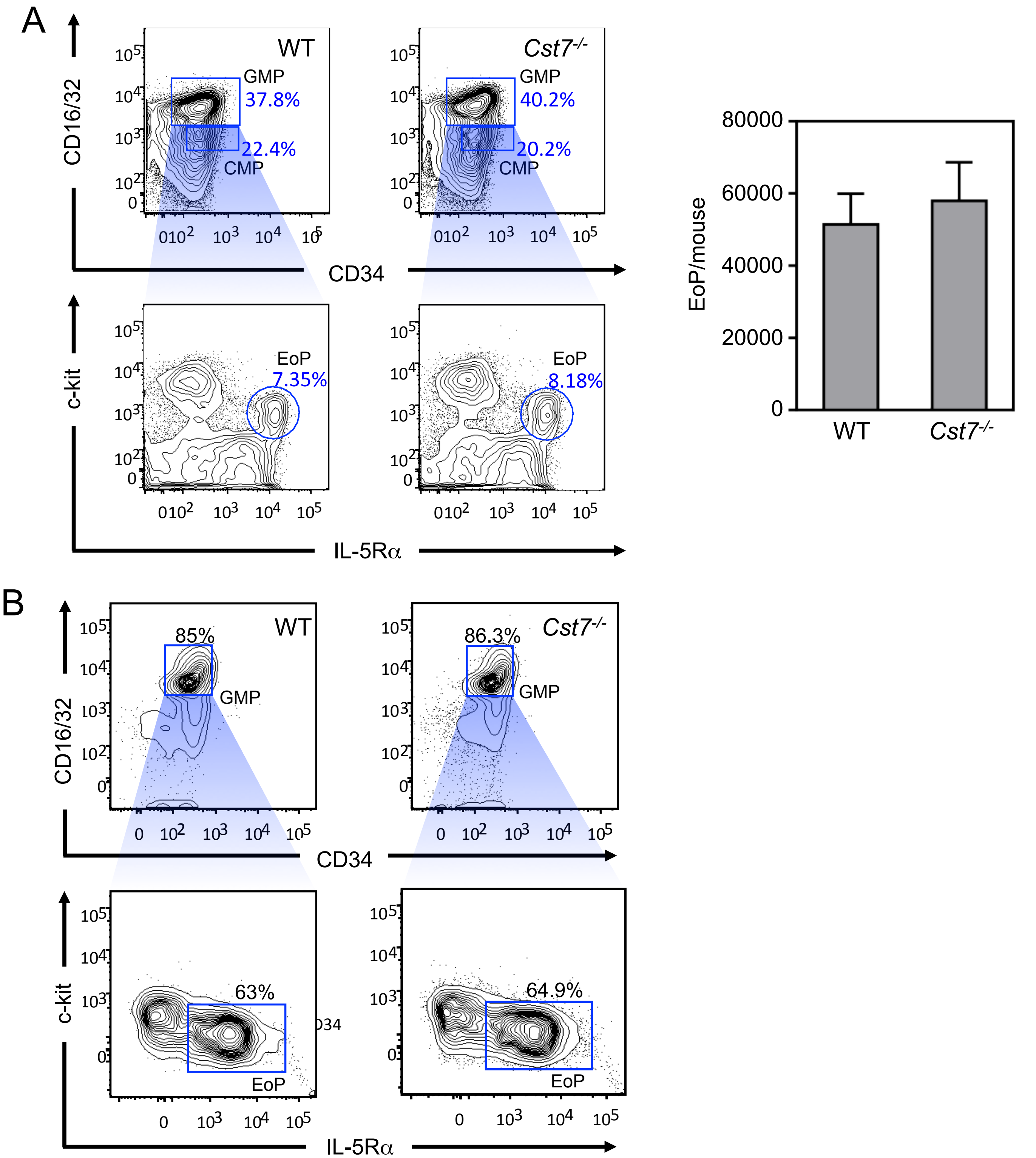

Figure S4, related to Figure 2: Eosinophil precursors are unaffected in CF null mice. (A) Lineage-positive bone marrow cells were excluded from analysis by staining with CD3, CD4, CD8α, CD19, B220 and Gr1 and common myeloid precursors (CMP; CD34^{+} CD16/32^{mid}) and granulocyte-macrophage precursors (GMP; CD34+ CD16/32^{high}) were identified from the remaining fraction. Similar proportions of c-kit^{mid} IL-5Rα^{+} eosinophil precursors (EoP) were detected within the GMP populations of wild type and *Cst7-/-* mice. Histogram: Total EoP count per femur (mean ±SEM, n = 4 mice).
(B) Equivalent proportions of GMP & EoP in bone marrow cultures from wild type and *Cst7-/-* mice expanded for 4 days in Flt3-L & SCF-1.

Figure S4

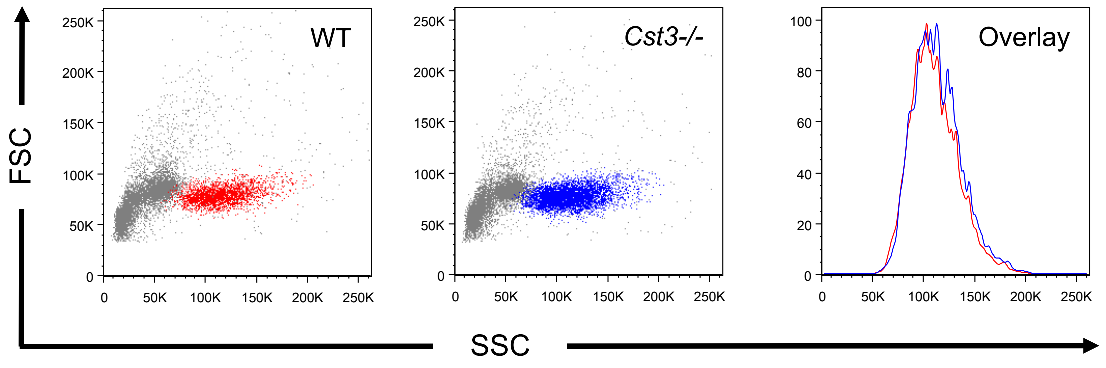

Figure S5, related to Figure 2: Cystatin C is not required for eosinophil granule integrity. Eosinophils were identified in peripheral blood from age-matched wild type or cystatin C null mice (*Cst3-/-*) by Siglec F staining. Granularity was normal in the absence of cystatin C (side scatter overlays).

Figure S5

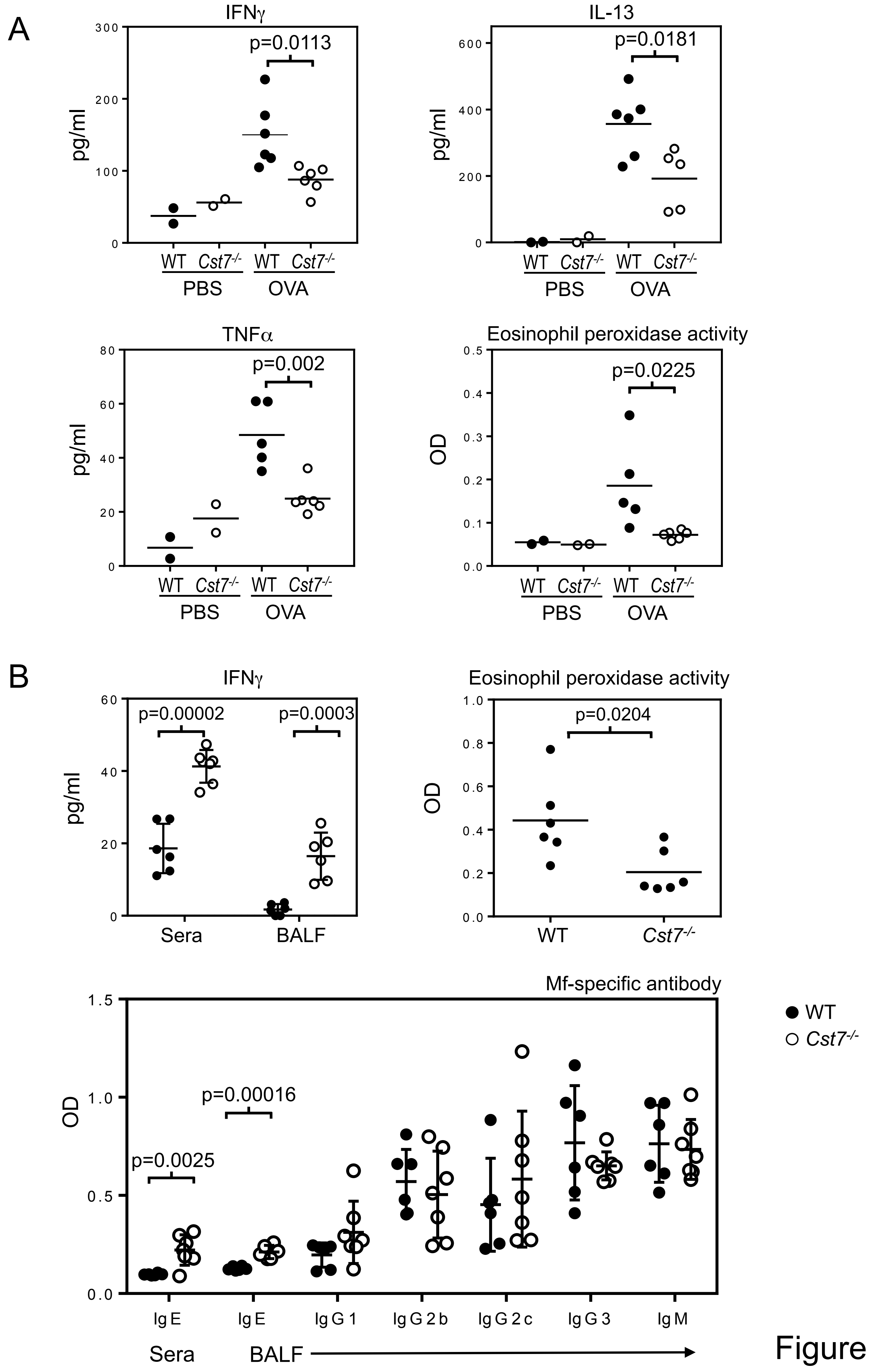
A
IFNγ
p=0.0113
pg/ml
WT Cst7-/- WT Cst7-/-
PBS OVA
IL-13
p=0.0181
TNFα
p=0.002
Eosinophil peroxidase activity
p=0.0225
OD
B
IFNγ
p=0.00002
p=0.0003
Sera
BALF
Eosinophil peroxidase activity
p=0.0204
WT
Cst7-/-
Mf-specific antibody
p=0.0025
p=0.00016
Ig E
Ig E
Ig G 1
Ig G 2 b
Ig G 2 c
Ig G 3
Ig M
Sera
BALF
WT
Cst7-/-

Figure S6

Figure S6, related to Figure 7: CF null mice display altered inflammatory parameters during eosinophilic inflammation.

(A) Levels of IFNγ, IL-13, TNFα and Epx were measured in cell-free BAL fluid from mice sensitized to OVA and sacrificed after 5 days of aerosol exposure (acute protocol). Values for individual mice plus means for each group are shown.

(B) IFNγ and Mf-specific antibody titres were quantitated in BAL fluid & sera collected 28 days after infection with *B. malayi* Mf. Reduced Epx activity was also detected in the cell-free BALF. Data represent values for individual mice plus means for each group.

Table S1, related to main text Figure 6: Identification of eosinophil granule proteins by LC-MS/MS.

Granules were released from *in vitro* cultured bone marrow eosinophils by mechanical lysis followed by intermediate-speed centrifugation (10 000g; 30min) of the post-nuclear supernatant (Ackerman et al, *J. Immunol* 1983 (131): p2977-82). Washed granule pellets were solubilized in RapiGest (Waters Ltd, UK), reduced, alkylated and digested with trypsin. 1.5µg of granule lysates were analysed with an Orbitrap Velos Pro mass spectrometer coupled to an Ultimate 3000 UHPLC system and protein identification and label-free quantification (LFQ) were performed using MaxQuant Version 1.3.0.5. Granules from 3 independent eosinophil preparations were analysed and 2498 proteins were identified. Protein IDs are presented in decreasing order of mean LFQ intensity (≅ abundance).
